# Syndrome of Inappropriate Antidiuretic Hormone Secretion Following Ayahuasca Use in a Satanic Ritual: A Case Report

**DOI:** 10.7759/cureus.24310

**Published:** 2022-04-20

**Authors:** Maryam Bayat Mokhtari, Moein Bayat Mokhtari, Blanca Z Rodriguez, Ting Yu Yen, Ivan D Rodriguez

**Affiliations:** 1 Department of Family Medicine, Larkin Community Hospital, South Miami, USA; 2 Department of Internal Medicine, Lenox Hill Hospital, New York City, USA; 3 Department of Internal Medicine, Larkin Community Hospital, South Miami, USA

**Keywords:** hallucinogens, adh, hyponatremia, siadh, ayahuasca

## Abstract

Ayahuasca is a psychedelic blend originating from South America that has been used for hundreds of years by local tribes in ritualistic ceremonies. For the last few decades, its usage has expanded to North America in various mindfulness retreats that can benefit from its hallucinogenic properties. In this case report, our patient had attended a satanic ritual where he had consumed copious amounts of Ayahuasca over three days. Upon his return, he continued having demonic hallucinations along with paranoid delusion with increasing bouts of nausea and vomiting. He was brought in by first responders due to suicidal ideations, and shortly after arrival, he became unresponsive with seizure-like activity. The patient was evaluated to be comatose with a Glasgow Coma Scale (GCS) score of 3, and upon securing the airway, was transferred to the critical care unit. Laboratory results showed a case of hypoosmolar hyponatremia secondary to syndrome of inappropriate antidiuretic hormone secretion (SIADH), and following the supportive care, he was able to recover within four days of admission. We theorize that due to the similarity in the pharmacodynamics of the active compound of Ayahuasca, and drugs such as monoamine oxidase inhibitors (MAOI) and 3,4-methylenedioxymethamphetamine (MDMA), it can result in SIADH in its users.

## Introduction

Ayahuasca is a psychedelic concoction prepared from two species of plants (*Banisteriopsis caapi *and* Psychotria viridis*) originating from aboriginal tribes of South America. Ayahuasca is used by the local tribes to create a harmonious connection between body and soul to treat physical and psychological ailments through its hallucinogenic properties [1]. In last few decades, with the rise of internet, use of Ayahuasca has spread to the United States in various rituals. It is considered a natural way to help with depression and other psychological issues through increasing one’s mindfulness and self-awareness [2]. Studies have shown the main psychoactive ingredients of Ayahuasca to be alkaloids such as β-carbolines and dimethyltryptamines (DMT), which cause hallucinations through acting as monoamine oxidase inhibitor and activating the serotonin receptors [1]. Here we demonstrate how these active ingredients led to syndrome of inappropriate antidiuretic hormone secretion (SIADH) and, in turn, hypoosmolar hyponatremia.

## Case presentation

A 56-year-old South American man with past medical history of hypertension, diabetes mellitus and hyperlipidemia was brought into our facility by Miami Dade fire and rescue under the provisions of Bakers Act. He had returned from Orlando a day prior to admission, where he had attended a Satanic ritual to help with his self-diagnosed depression. During this retreat, the patient had consumed copious amounts of Ayahuasca concoction over a period of three days leading to multiple episodes of hallucinations. Since his return, he had been having paranoid delusions of being followed by two black pickup trucks wanting to kidnap him. Additionally, the patient hallucinated demons and angels to whom he actively tried praying to. Shortly after, he developed significant abdominal discomfort with worsening nausea and vomiting episodes throughout the day. Once he began verbalizing suicidal ideation, the emergency medical services were contacted by his daughter.

**Figure 1:**
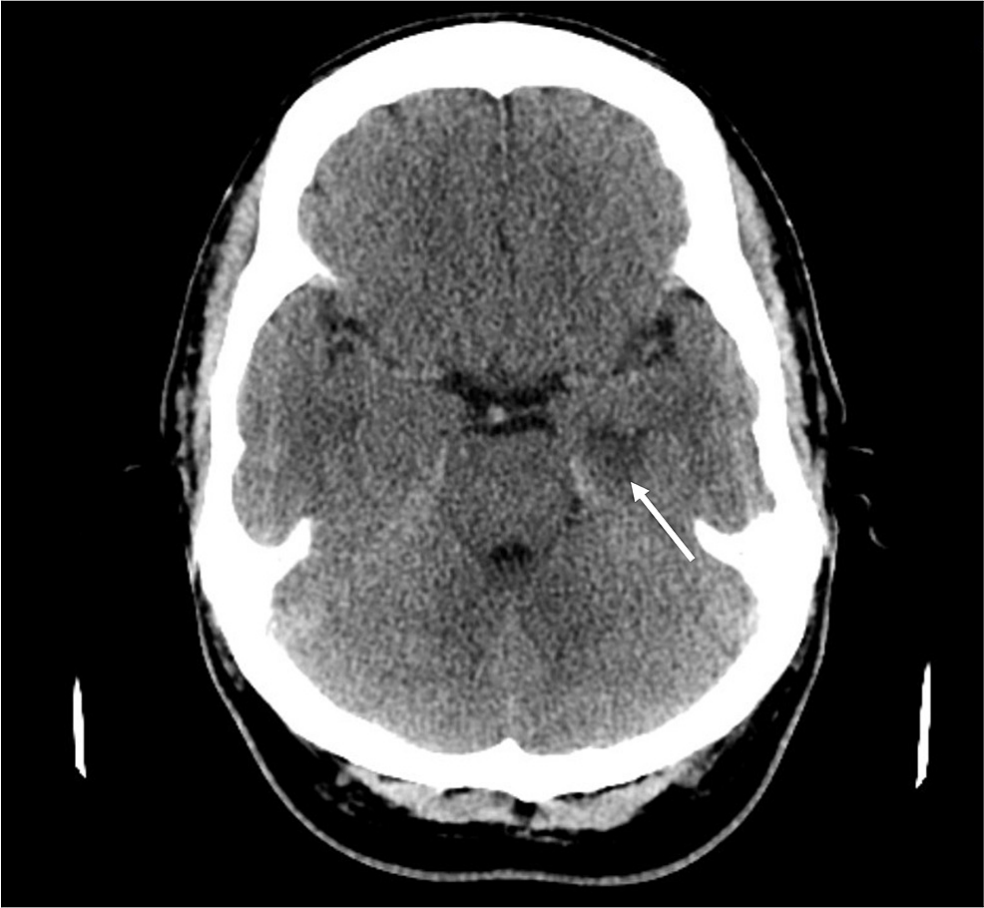
Axial computed tomography (CT) of the brain showing asymmetric hypoattenuation at the left medial temporal lobe indicating edema (white arrow)

On initial presentation at the emergency department, the patient was alert and oriented to person and place despite presenting with bizarre behavior. The suicidal screen was negative at the time, and the only abnormal findings were hypertensive urgency (235/141 mmHg) and tachycardia (101 beats per minute (bpm)). During history taking, the patient suddenly became unresponsive, was unable to produce any sound, developed fecal and urinary incontinence, and exhibited upper extremity twitching consistent with seizure activity. Rapid response was called, and the patient was evaluated to have a GCS score of 3. To protect his airway, he was intubated, sedated, and mechanically ventilated. Nitroprusside intravenous (IC) drip was started to assist with hypertensive urgency, and the patient was transferred to the intensive care unit (ICU). On examination, the pupils were fixed at 3 mm non-reactive to light, and upper extremity twitching/seizure activity continued despite administration of IV levetiracetam 500 mg. Brain computed tomography (CT) without contrast taken shortly after the rapid response showed vague asymmetric hypoattenuation at the left medial temporal lobe with nonspecific findings that could represent edema (Figure 1). No signs of ischemia were observed.

About an hour after transfer to ICU, the patient became transiently agitated despite being sedated with propofol 50 mcg/kg/min IV drip and midazolam, during which episode he had regained the ability to move upper and lower extremities. Further physical examination showed organized pattern of intentional skin lesions on right upper extremity in circular fashion. It was later found that these were created as part of the ritual to administer Kambo, a poisonous substance derived from secretions of *phyllomedusa bicolor*, better known as giant leaf frog [3]. Poison control was contacted to obtain information, and they confirmed the presentation to be potentially due to Ayahuasca toxicity. Labs were obtained during initial hours of admission, and they were significant for serum sodium of 122 mEq/L, serum osmolality of 246 mOsm/Kg, urine sodium of 59 mEq/L and urine osmolality of 285 mOsm/Kg. Additionally, the patient had electrolyte deficiency in the form of hypokalemia and hypomagnesemia, in addition to acid-base imbalance in the form of respiratory alkalosis. The patient's toxicology report on admission was negative for all routinely tested substances, including amphetamines, cocaine and opiates. Appropriate supportive measures were taken to treat these conditions. Table 1 summarises the patients lab findings from the time of admission to discharge.

**Table 1:** The patient laboratory findings from the time of admission (day 1) to discharge (day 4) BUN: blood urea nitrogen, AST: aspartate aminotransferase, ALT: alanine aminotransferase

Lab	Day 1	Day 2	Day 3	Day 4	Reference range
Glucose	139	96	84	94	70-99 mg/dL
BUN	8	11	9	8	6-24 mg/dL
BUN/Creatinine	11	11	10	9	-
Sodium, serum	123	134	139	137	135-145 mEq/L
Potassium	2.6	3.9	3.7	3.4	3.5-5.2 mEq/L
Chloride	87	106	104	102	96-106 mEq/L
Osmolality, serum	248.7	267.5	275.4	271.9	275-295 mOsm/kg
Albumin, serum	5.0	3.1	3.6	3.7	3.4-5.4 g/dL
AST	32	13	37	27	8-31 U/L
ALT	33	17	25	24	7-56 U/L
Urine osmolality, mOsm/Kg	285	-	-	-	-
Urine sodium, mEq/L	59	-	-	-	-

The patient started to significantly improve over the following day and was extubated. He remained hypertensive with a blood pressure steadily around 180/106 mmHg. He was eventually transitioned to oral amlodipine 10 mg, oral losartan 50 mg, and oral carvedilol 6.25 mg, which proved to be effective for his blood pressure control. The following day he was transferred to the general medicine floor with complete resolutions of symptoms. He was followed by psychiatry and neurology, who collectively agreed on poisoning to be the sole reason for his psychiatric and physiological manifestations.

The patient’s laboratory findings and clinical presentation were classical for SIADH. This patient’s admission laboratory findings clearly demonstrated hyponatremia (122 mEq/L), reduced serum osmolality (246 mOsm/Kg), increased renal sodium excretion (59 mEq/L) and an increased urine osmolality (285 mOsm/Kg). Additionally, on clinical examination the patient demonstrated no clinical evidence of volume depletion such as hypotension and had normal skin turgor indicating the patient was in a state of euvolemia. Moreover, the patient’s normal liver function tests, normal toxicology screen and kidney function tests exclude any other causes of hyponatremia. Finally, the patient’s condition and lab findings improved following restriction of fluids once the patient was stabilized in the ICU. These findings fulfil the Schwartz and Bartter clinical diagnostic criteria for SIADH [4]. He was discharged on day four of admission and was scheduled to follow up with an MRI as an outpatient.

## Discussion

SIADH is an endocrine disorder of excessive secretion of antidiuretic hormone (ADH) from pituitary gland. This increased ADH secretion has been explored thoroughly in literature and have been associated with medications/drugs, central nervous system conditions, chronic disease amongst various other etiologies, including malignancies [5]. Clinically, patients with SIADH present with hyponatremia that has been resulted due to excessive water retention by kidneys. Furthermore, this excess retention is expected to lower serum osmolality with relative increase in urine osmolality and salt excretion. Lastly, due to retention of water, these patients present with a euvolemic status [6]. Our patient who suffered from Ayahuasca overdose perfectly fits the above criteria by having severe hyponatremia and low serum osmolality in addition to increased urine osmolality and sodium excretion. Severe hyponatremic encephalopathy in this patient could explain many of his acute neurological presentations, including altered consciousness, seizures and CT findings indicating potential edema. 

One common etiology of SIADH that is also of particular interest are medications. Studies have shown certain classes of antidepressants and recreational drugs, particularly monoamine oxidase inhibitors (MAOI) and 3,4-methylenedioxymethamphetamine (MDMA), to cause SIADH in patients [5]. Studies have shown that active ingredients in Ayahuasca include DMT. These ingredients not only have MAOI properties but also function identically to MDMA by acting as serotonin receptor agonists [1]. Hence, we theorize that due to this similarity in pharmacodynamic actions, Ayahuasca overdose is potentially capable of also causing SIADH in users. However, it is important to note that a limitation is the patient's use of Kambo, poisonous secretion of giant leaf frog, in addition to Ayahuasca [3]. This substance is derived from poisonous secretions of giant leaf frogs. Use of Kambo in rituals is usually accompanied by drinking large amounts of water. Also, Kambo has a physiologic ability to alter normal hunger and thirst responses in individuals [7]. Therefore, this can result in excessive drinking habits, potentially resulting in dilutional hyponatremia. However, in the case of primary polydipsia, it is generally expected to see a low urine osmolality which was not seen in this patient. Though the potential interaction between these two compounds is difficult to assess in one individual case.

## Conclusions

This case report suggests that Ayahuasca could be a potential cause of hypoosmolar hyponatremia secondary to SIADH, which physicians should be aware of. It is important to obtain a detailed history of substance use in likely patients and be prepared for potential complications that may arise from hyponatremia. Recognizing the symptoms of hyponatremia and a timely intervention can greatly improve the patient prognosis. Given the novel nature of this case report and lack of adequate knowledge about Ayahuasca, we suggest further investigation on this compound and its pharmacological properties in human body. 
